# Medizin und Medien – Symbiose oder Konflikt?

**DOI:** 10.1007/s00103-020-03262-0

**Published:** 2020-12-16

**Authors:** Suzan Fiack, Uwe Koch-Gromus, Joseph Kuhn, Carola Lübbing-Raukohl

**Affiliations:** 1 Bundesinstitut für Risikobewertung, Max-Dohrn-Straße 8–10, 10589 Berlin, Deutschland; 2 Institut und Poliklinik für Medizinische Psychologie, Universitätsklinikum Hamburg-Eppendorf, Martinistraße 52, 20246 Hamburg, Deutschland; 3 GE 4.2 Gesundheitsberichterstattung, Sozialmedizin, Öffentlicher Gesundheitsdienst, Bayerisches Landesamt für Gesundheit und Lebensmittelsicherheit, Veterinärstr. 2, 85764 Oberschleißheim, Deutschland; 4 Paul-Ehrlich-Institut, Bundesinstitut für Impfstoffe und biomedizinische Arzneimittel, Paul-Ehrlich-Str. 51–59, 63225 Langen, Deutschland

„Ich habe Besseres zu tun“, schrieb der Virologe Christian Drosten im Frühjahr 2020 auf Twitter, als es um eine kurzfristige Medienanfrage ging. Er entfachte mit seinem Tweet einmal mehr die Diskussion um die Rolle von Medizin und Medien in unserer Gesellschaft. Denn nicht nur in Coronazeiten stellt sich die Frage, wie das Zusammenspiel zwischen Wissenschaft und Journalismus gelingen kann. Handelt es sich um eine Erfolg versprechende Symbiose oder einen nicht lösbaren Konflikt? Dieser Frage gehen wir in unserem Themenheft „Medizin und Medien“ nach. Dabei befassen wir uns mit den medizinischen Informationsangeboten für die breite Öffentlichkeit in den klassischen Medien, im Internet und in den sozialen Medien.

Mit der Digitalisierung eröffnen sich für Medizin und Wissenschaft neue Möglichkeiten, Inhalte direkt mit der breiten Öffentlichkeit zu teilen. Diesen Medienwandel beschreiben *Daube et al*. in ihrem Übersichtsbeitrag und sehen durchaus Chancen für eine symbiotische Beziehung zwischen Wissenschaft und Medien – nicht nur angesichts der COVID-19-Pandemie. Viele Menschen verfügen häufig nicht über ausreichend Wissen, um Informationen korrekt einordnen zu können. Journalistische Beiträge können helfen, indem sie Originalaussagen der Medizin und Wissenschaft in den sozialen Netzwerken laienverständlich formulieren und einbetten.

Inwieweit werden medizinische Informationen vor der Weitergabe in den Medien wissenschaftlich geprüft? *Daube et al*. schlagen eine evidenzorientierte Berichterstattung vor. Diese soll auf den aktuellen Erkenntnissen beruhen und dabei explizit auch auf wissenschaftlich noch ungesicherte Befunde und Forschungslücken eingehen. Auch *Anhäuser et al.* erscheinen Qualitätsstandards notwendig. Sie haben einen modularisierten Kriterienkatalog für guten Medizinjournalismus erarbeitet. Dieser „Medien-Doktor“ soll zum Beispiel helfen, sorgfältig recherchierte Berichterstattung von interessengeleiteten Beiträgen oder gar Fake News besser zu unterscheiden.

Serien und Quizshows erreichen meist mehr Menschen als klassische Gesundheitssendungen. Wie seltene Erkrankungen dadurch mehr Aufmerksamkeit in der Bevölkerung erlangen erläutern *Schäfer et al.* unter anderem am Beispiel der TV-Sendungen „Dr. House“ und „Hirschhausens Quiz des Menschen“. Selbst Medizinstudierende können daraus etwas lernen.

Auch Krankheiten mit hoher Prävalenz, wie Diabetes mellitus und Depression, sind nur selten Hauptthema in den Medien. Zudem wird eine medikamentöse Therapie häufiger thematisiert als niedrigschwellige Maßnahmen oder Möglichkeiten der Prävention. *Reifegerste et al.* schlagen daher vor, dass Kommunikatorinnen und Kommunikatoren diese Aspekte noch stärker berücksichtigen.

Nicht minder spannend ist die Frage, wie die Gesundheitskommunikation bestimmte Zielgruppen erreichen kann. *Stehr et al.* kommen zu dem Schluss, dass die Zielgruppe 65+ am besten über traditionelle Massenmedien erreicht wird. Auf Twitter oder Facebook erreichen aber inzwischen auch Einzelpersonen größere Zielgruppen. *Lindemann et al. *beschreiben Meinungsführerinnen und -führer als Personen, die in ihrem sozialen Umfeld die Meinungen, Einstellungen oder das Verhalten von anderen beeinflussen können. Sie sind daher eine zentrale Zielgruppe für die Kommunikation von Gesundheitsthemen.

*Peter et al.* geben einen Überblick über die komplexe Rolle von Medien im Rahmen einer Essstörung und zeigen Lücken in der kommunikationswissenschaftlichen Forschung zum Thema auf. Lückenhaft sind allerdings auch die Daten für eine systematische Analyse zum Thema „Homöopathie in den Medien“. Daher nähern sich *Grams et al.* dem Thema essayistisch. Ihre These: Die Medienberichterstattung zur Homöopathie bewegt sich im Vergleich zu anderen medizinischen Themen auffallend häufig außerhalb des evidenzbasierten wissenschaftlichen Kontextes.

Die Medien hatten 2020 „nichts Besseres zu tun“, als über COVID-19 zu berichten. Dies beleuchtet unter anderem der Ausblick von *Volker Stollorz* am Ende des Heftes. Er zeigt nicht nur, dass das Volumen der Berichterstattung zu COVID-19 ein sehr hohes Niveau erreicht hat, sondern auch, dass erst die Massenmedien dieses zu einem der konzentriertesten Weltereignisse aller Zeiten gemacht haben. Der Autor zieht zudem eine wichtige Schlussfolgerung aus der Pandemieberichterstattung: Guter und professioneller Wissenschaftsjournalismus ist in der Demokratie essenziell.

Der wissenschaftliche Diskurs kennt nicht nur die binären Zustände „wahr“ und „falsch“, sondern führt in vielen Fällen eher mäandrisch zu einem Zugewinn an Wissen. Vermittlungsagenturen wie der Wissenschaftsjournalismus oder auch die Gesundheitsberichterstattung (siehe dazu Heft 9/2020), die helfen, Daten zu verstehen und einzuordnen, sind in einer Wissensgesellschaft auch vor diesem Hintergrund notwendig. Ihre Funktion zu reflektieren, ist dann wiederum eine (medien-)wissenschaftliche Aufgabe. Einige Aspekte dazu haben wir in diesem Heft aufgegriffen, und in diesem Sinne wünschen wir Ihnen eine anregende Lektüre.

